# Estimating the Disease Burden of 2009 Pandemic Influenza A(H1N1) from Surveillance and Household Surveys in Greece

**DOI:** 10.1371/journal.pone.0020593

**Published:** 2011-06-09

**Authors:** Vana Sypsa, Stefanos Bonovas, Sotirios Tsiodras, Agoritsa Baka, Panos Efstathiou, Meni Malliori, Takis Panagiotopoulos, Ilias Nikolakopoulos, Angelos Hatzakis

**Affiliations:** 1 National and Kapodistrian University of Athens, Athens, Greece; 2 Hellenic Centre for Diseases Control and Prevention, Athens, Greece; 3 National Health Operations Centre, Ministry of Health and Social Solidarity, Athens, Greece; Dana-Farber Cancer Institute, United States of America

## Abstract

**Background:**

The aim of this study was to assess the disease burden of the 2009 pandemic influenza A(H1N1) in Greece.

**Methodology/Principal Findings:**

Data on influenza-like illness (ILI), collected through cross-sectional nationwide telephone surveys of 1,000 households in Greece repeated for 25 consecutive weeks, were combined with data from H1N1 virologic surveillance to estimate the incidence and the clinical attack rate (CAR) of influenza A(H1N1). Alternative definitions of ILI (cough or sore throat and fever>38°C [ILI-38] or fever 37.1–38°C [ILI-37]) were used to estimate the number of symptomatic infections. The infection attack rate (IAR) was approximated using estimates from published studies on the frequency of fever in infected individuals. Data on H1N1 morbidity and mortality were used to estimate ICU admission and case fatality (CFR) rates. The epidemic peaked on week 48/2009 with approximately 750–1,500 new cases/100,000 population per week, depending on ILI-38 or ILI-37 case definition, respectively. By week 6/2010, 7.1%–15.6% of the population in Greece was estimated to be symptomatically infected with H1N1. Children 5–19 years represented the most affected population group (CAR:27%–54%), whereas individuals older than 64 years were the least affected (CAR:0.6%–2.2%). The IAR (95% CI) of influenza A(H1N1) was estimated to be 19.7% (13.3%, 26.1%). Per 1,000 symptomatic cases, based on ILI-38 case definition, 416 attended health services, 108 visited hospital emergency departments and 15 were admitted to hospitals. ICU admission rate and CFR were 37 and 17.5 per 100,000 symptomatic cases or 13.4 and 6.3 per 100,000 infections, respectively.

**Conclusions/Significance:**

Influenza A(H1N1) infected one fifth and caused symptomatic infection in up to 15% of the Greek population. Although individuals older than 65 years were the least affected age group in terms of attack rate, they had 55 and 185 times higher risk of ICU admission and CFR, respectively.

## Introduction

Soon after the identification of the first cases infected with the new influenza A(H1N1) strain in Mexico and USA in March-April 2009, the virus spread rapidly around the world. H1N1 influenza activity initially peaked in the United States during May and June 2009 and a second wave occurred during the fall with activity peaking during the second week in October [1]. In Europe, an initial spring/summer wave of transmission appeared in most countries and was followed by a sharper autumn/winter wave of infection affecting all countries [2]. The first case of influenza A(H1N1) in Greece was reported on May 18, 2009 and the epidemic continued up to April 2010 when the last cases were recorded.

A major challenge in influenza pandemics is to estimate the proportion of the population that was symptomatically infected and to draw conclusions concerning the age-specific severity of the infection. Estimating the attack rate of an influenza pandemic using existing influenza surveillance systems is challenging since only a portion of symptomatic cases seek medical care and only a small number of those seeking medical care are usually tested. During the 2009 influenza A(H1N1) pandemic, several reports have attempted an assessment of the associated burden and provided estimates of hospitalisations, ICU admissions or death rates using the number of laboratory-confirmed cases or the size of the general population as denominator [3]–[6]. Few studies have endeavored to estimate the attack rate of influenza A(H1N1) or to provide more accurate estimates of morbidity and mortality rates [7]–[13].

A proposed method, complementary to existing surveillance, in ascertaining the true incidence of influenza-like illness is repeated population-based telephone surveys [14], [15]. Telephone surveys have been used during the 2009 pandemic to assess attitudes and perceptions concerning influenza [16], [17] as well as in the past to estimate the prevalence of seasonal influenza or of influenza-like illness [18], [19]. In Greece, repeated cross-sectional nationwide telephone surveys of 1,000 households were conducted for 25 consecutive weeks, and covered the major epidemic wave during the autumn and early winter of 2009. The aim of the present study was to estimate the overall and age-specific incidence as well as the attack rate of influenza A(H1N1) in the Greek population by combining the data collected by the telephone surveys with data from virologic surveillance. Secondary aims were to provide estimates of the case fatality rate (CFR) as well as of the burden of disease of health care services. The collected data allowed to trace the spread of influenza A(H1N1) in the population over time and to identify the most affected age groups.

## Methods

### Ethics

According to the decision of the Board of Directors of the Hellenic Center for Disease Control and Prevention and of Athens University, the household telephone surveys were deemed public health practice and not human subjects research and therefore did not require ethics approval by the Institutional Review Board of Athens University Medical School. Contacted persons gave verbal consent to participate in a telephone survey conducted by the University of Athens.

### Telephone surveys

Data on the incidence of influenza-like illness (ILI) were obtained by repeated cross-sectional nationwide telephone surveys of 1,000 households in Greece on a weekly basis, starting the last week of August 2009 (Week 35 of 2009) until mid-February 2010 (Week 6 of 2010) (total duration, 25 weeks). Proportional quota sampling was used each week to ensure that selected households were representative of Greek households, with quotas based on household size and urban/rural location. A non-random adult from each household was asked to participate to a survey. To reduce selection bias, the participants were initially asked to participate to a survey performed on behalf of the University of Athens without mentioning that the survey was related to influenza A(H1N1). In case the person accepted to participate, he/she was asked to provide information on several items including the occurrence of each of the following symptoms related to influenza during that particular week for each household member: fever (37.1–38.0°C or >38°C), cough, sore throat, runny nose. Other data, including perceptions and beliefs concerning influenza and vaccination, were also collected [20], [21] (more details in **Supporting Information S1**).

### Estimating the incidence and attack rate of influenza A(H1N1)

The definition of ILI usually includes the presence of fever >38°C and cough or sore throat (ILI-38). Due to the limited sensitivity of ILI-38 for symptomatic influenza infection [22], [23], alternative definitions of ILI and acute respiratory illness (ARI) were also used. Thus, we denote as ILI-37 the presence of fever 37.1–38°C and cough or sore throat, as ARI-38 the presence of any two of the following symptoms: fever >38°C, cough, sore throat, and runny nose, and as ARI-37 the presence of any two of the following symptoms: fever 37.1–38°C, cough, sore throat and runny nose.

**Figure 1:**
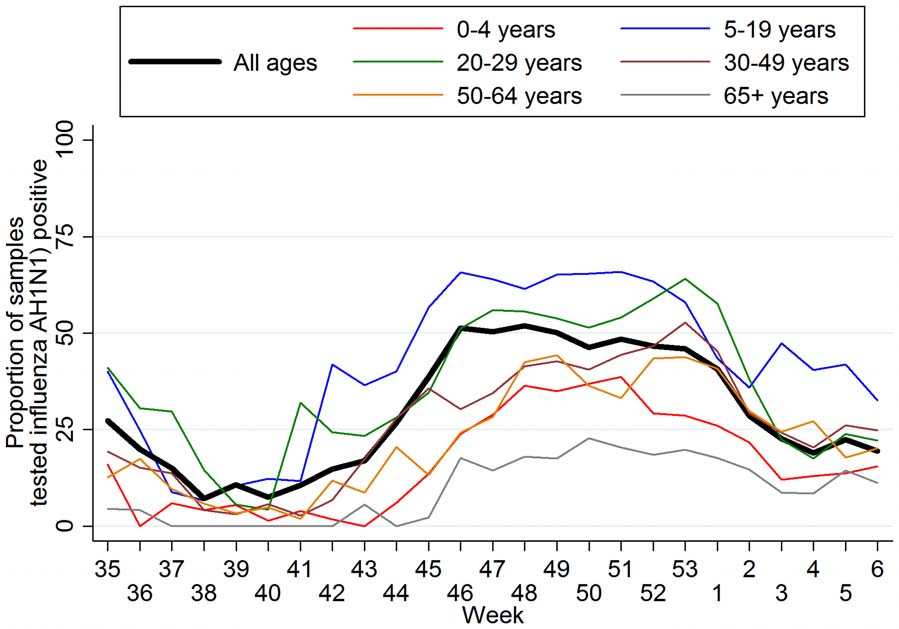
Proportion of samples testing positive for 2009 pandemic influenza A(H1N1) by RT-PCR over time. All samples (15,494 samples tested positive out of a total of 37,960 samples) and according to age group (number testing positive/total tested: 706/2865, 4431/7719, 1966/4194, 2442/6996, 996/3145, 380/2779 for the age groups 0–4, 5–19, 20–29, 30–49, 50–64, and 65+ years, respectively).

The estimate of the incidence of symptomatic influenza A(H1N1) was based on the weekly age-specific ILI rate data, obtained from the phone surveys, and the data on the proportion of samples testing positive for H1N1 out of the total number of samples sent to laboratories from hospitals in the corresponding weeks. We assumed 90% sensitivity for the RT-PCR test [24]. We have applied the age-specific estimates of the proportion of samples testing positive on the corresponding age-specific ILI rates to obtain the number of H1N1 cases as the proportion testing positive varied largely according to age (**Figure 1**). Thus, for a particular week *i* and age group *j* where *ILI_ij_*: the estimated number of new ILI cases per 100,000 population and *PROP_i_*_j_: the proportion of samples testing positive for H1N1, the incidence of symptomatic H1N1 cases was estimated as: 
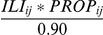
 per 100,000 population per week. Alternative incidence estimates were obtained using the number of ILI-38, ILI-37, ARI-38 and ARI-37 cases in this formula. A refinement of this method would be possible by taking into account of the variability of the estimates of *ILI_ij_* and *PROP_i_*_j_ obtained by the weekly samples.

The number of infections, both symptomatic and asymptomatic, was approximated using the estimates provided by two studies on the frequency of fever among infected individuals [22], [23]. According to Carrat et al, fever >37.8°C was observed in 37% of A/H1N1 infections [22]. Similarly, Cao et al reported that 36% of infected individuals experienced fever >38.0°C [23]. Thus, the estimated incidence of symptomatic H1N1 infection (based on ILI-38 case definition) was divided by 36% to obtain the corresponding estimate for H1N1 infection.

The clinical attack rate (CAR) and the infection attack rate (IAR) were estimated as the cumulative incidence of symptomatic infections and of all estimated infections, respectively, during the 25-week period covered by the phone surveys.

To account for the sampling design, the *svy* commands (STATA, StataCorp LP) were employed with the design properly specified in order to obtain estimates and their standard errors.

### Estimating the burden of disease (GP consultations, hospital admissions, ICU admissions, case fatality rates)

Data collected from the phone surveys concerning GP consultations and hospital visits/admissions (**Supporting Information S2**) were used to estimate the proportion of symptomatic cases seeking medical care from their GPs/hospital emergency departments or being hospitalized.

Surveillance data on the number of laboratory confirmed influenza A(H1N1) cases that were admitted to ICU or died were collected as described elsewhere [25]. Briefly, all hospital administrations in Greece were asked to report patients admitted with confirmed or suspected cases of 2009 H1N1 three times weekly. On a daily basis, investigators from the National Health Operations Centre of the Ministry of Health made follow-up telephone calls to the physicians of all patients with confirmed cases of 2009 influenza A(H1N1) who were admitted to an ICU. Data collection on deaths was performed by the Hellenic Centre for Diseases Control and Prevention in collaboration with the National Health Operations Centre from a network that included all state and private hospitals in the seven semiautonomous regional health authorities of Greece.

ICU admission rates and case fatality rates (CFR) were estimated per symptomatic cases as well as per estimated H1N1 infections. The estimates of H1N1 case numbers and the data collected on the number of deaths and ICU admissions up to week 6 of 2010 were used to calculate age-specific and overall ICU admission rates and CFR. To allow the comparability of these results with estimates from other studies, rates per symptomatic cases were calculated using the number of symptomatic H1N1 cases obtained based on the standard ILI-38 definition. To account for the lag between the onset of symptoms and the occurrence of these events, the cumulative number of cases one and two weeks before week 6 of 2010 for the estimate of ICU admission rates and CFR, respectively, were used [11].

## Results

### Response rates and sample characteristics

Overall, 81,268 households were contacted with members eligible to participate during the 25 weeks. Of these, 25,012 agreed to participate (response rate: 30.8%). Weekly response rates ranged from 25.7% to 34.5% throughout the study.

The weekly samples of 1,000 households included in the surveys were located in Athens (main metropolitan area of Southern Greece, 40% of households), Thessaloniki (main metropolitan area of Northern Greece, 10% of households), and other urban (25%), semi-urban (10%), and rural (15%) areas. In total, there were 72,201 members in the participating households. The average household size was 2.9 persons. Females accounted for 51.4% of the sample. Fourteen per cent, 11%, 57% and 18% of the sample were 0–14, 15–24, 25–64 and ≥65 years old, respectively. Age and gender distribution as well as the average household size of the sample were similar to that of the Greek population according to the data provided by the Hellenic Statistical Authority (**Supporting Information S3**).

### Incidence and attack rate of influenza A (H1N1)

The proportion of swabs collected from ILI cases that tested positive for H1N1 by RT-PCR according to age and over time is depicted in **Figure 1**. The prevalence of H1N1 positive samples was higher in the peak epidemic weeks and reached up to 65% in individuals aged 5–29 years. Furthermore, it was considerably lower among very young (0–4 years) and old (≥65 years) ILI cases.

**Figure 2:**
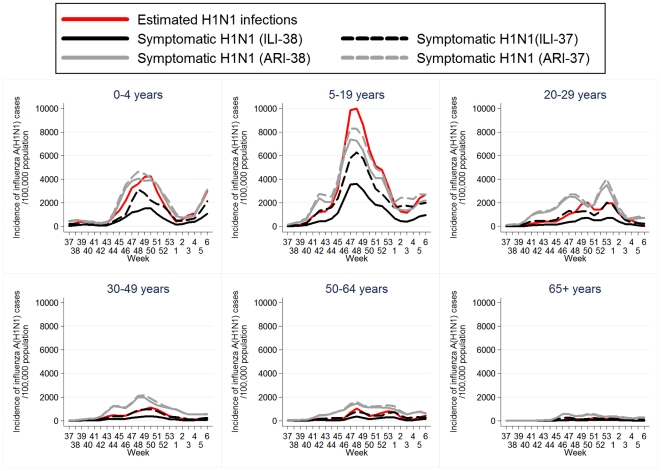
Age-specific incidence of 2009 pandemic influenza A(H1N1) per 100,000 population per week (3-week weighted moving average). For week *i* and age group *j*, the incidence of symptomatic H1N1 infection was estimated as: 
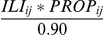
 per 100,000 population per week where *ILI_ij_*: the estimated number of new ILI cases per 100,000 population per week and *PROP_i_*_j_: the proportion of samples testing positive for H1N1. The number of estimated H1N1 infections was obtained by dividing the estimated number of symptomatic H1N1 cases (based on ILI-38 case definition) by 0.36 [22], [23]. (ILI-38: fever >38°C and cough or sore throat, ILI-37: fever 37.1-38°C and cough or sore throat, ARI-38: any two of fever >38°C, cough, sore throat and runny nose, ARI-37: any two of fever 37.1–38°C, cough, sore throat and runny nose).

**Figure 3:**
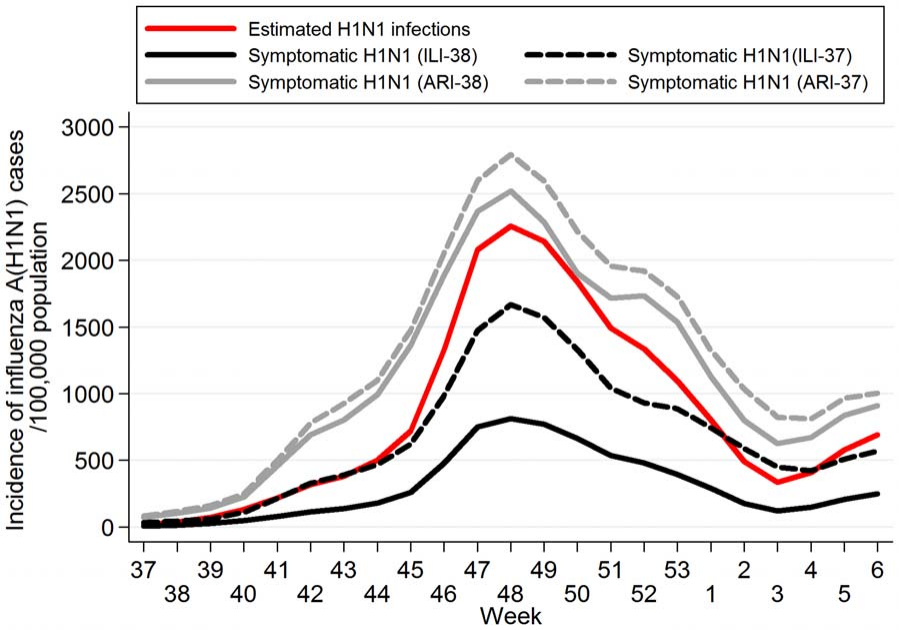
Incidence of 2009 pandemic influenza A(H1N1) per 100,000 population per week in Greece (3-week weighted moving average). (ILI-38: fever >38°C and cough or sore throat, ILI-37: fever 37.1–38°C and cough or sore throat, ARI-38: any two of fever >38°C, cough, sore throat and runny nose, ARI-37: any two of fever 37.1–38°C, cough, sore throat and runny nose).

The age-specific weekly incidence rates of ILI-38, ILI-37, ARI-38 and ARI-37 are depicted in **Figure S1**. A steady rise in the number of ILI and ARI cases was observed after week 37 of 2009 (September 7–13, 2009) in most age groups; particularly, among individuals aged 0–4 and 5–19 years. The age-specific incidence rate of H1N1 symptomatic cases and of estimated H1N1 infections is shown in **Figure 2**. The most affected age group was that of school children/adolescents 5–19 years old with a peak in the incidence occurring during week 48 of 2010 (approximately 3,300–5,800 new H1N1 symptomatic cases per 100,000 population per week, based on ILI-38 and ILI-37 definition, respectively). The overall incidence of ILI/ARI and of H1N1 in the Greek population is depicted in **Figure S2** and **Figure 3**, respectively. The epidemic peaked on week 48 (end of November-beginning of December 2009), with approximately 750–1,500 new H1N1 symptomatic cases per 100,000 population per week, based on ILI-38 and ILI-37 definition, respectively, and was followed by a sharp decline in the following weeks.

During the weeks 35/2009–6/2010, 7.1%–15.6% of the population in Greece was estimated to be symptomatically infected with H1N1, depending on ILI case definition (**Table 1**). When the number of H1N1 cases was estimated based on the number of ARI cases, the CAR was much higher (26.1%–29.5%) (**Table S1**). The IAR (95% CI) was estimated to be 19.7% (13.3%, 26.1%) (**Table 1**).

**Table 1:** 2009 Pandemic Influenza A(H1N1) Clinical Attack Rates (% symptomatically infected) and Infection Attack Rates (% infected) in Greece.

Age group	Clinical Attack Rate of Influenza A(H1N1)		Infection Attack Rate of Influenza A(H1N1)
	% (95% CI)		% (95% CI)
	Based on the number ofILI-38^a^ cases	Based on the number of ILI-37^b^ cases	
0–4 years	13.1	24.7	36.4
	(7.6–18.6)	(14.1–35.3)	(21.1–51.7)
5–19 years	27.4	53.7	76.2
	(15.5–39.4)	(34.3–73.0)	(43.0–100.0)
20–29 years	7.1	18.1	19.8
	(3.9–10.3)	(11.4–24.8)	(11.0–28.6)
30–49 years	3.1	8.1	8.7
	(1.7–4.5)	(4.8–11.5)	(4.8–12.5)
50–64 years	2.8	7.5	7.6
	(1.4–4.1)	(4.4–10.5)	(3.8–11.5)
65+ years	0.6	2.2	1.6
	(0.2–1.0)	(1.2–3.3)	(0.4–2.8)
**Greek population**	**7.1**	**15.6**	**19.7**
	**(4.8–9.4)**	**(11.5–19.7)**	**(13.3–26.1)**

Overall and Age-Specific 2009 Pandemic Influenza A(H1N1) Clinical Attack Rates (depending on the definition of influenza-like illness) and Infection Attack Rates (Week 35 of 2009 to Week 6 of 2010).

a ILI-38 : fever >38°C and cough or sore throat, ^b^ILI-37 : fever 37.1–38°C and cough or sore throat.

The age-specific estimates of the clinical attack rates are depicted in **Table 1**. School children and adolescents were the most affected group; 27.4%–53.7% were symptomatically infected with influenza A(H1N1), depending on ILI case definition. Children 0–4 years of age (13.1%–24.7%) and young adults 20–29 years of age (7.1%–18.1%) were also largely affected. CARs were markedly lower among individuals older than 30 years old (30–49 and 50–64 years old had CARs of 3.1%–8.1% and 2.8%–7.5%, respectively) and particularly, among individuals older than 64 years (0.6%–2.2%). Similar patterns were observed in the proportion of infected individuals in the different age groups (**Table 1**).

### ICU admission rates and case fatality rates

As of week 6 of 2010, there were 287 laboratory-confirmed cases admitted to ICU. The estimated ICU admission rate was 13.4/100,000 infected cases and 37.2/100,000 symptomatic cases. It varied widely according to age, with an estimated rate for individuals older than 65 years old that was 55-fold than that of individuals 5–19 years old (346.4 and 6.2 per 100,000 symptomatic cases, respectively) (**Table 2**).

**Table 2:** Overall and Age-Specific ICU Admission Rate per 100,000 symptomatic cases and per 100,000 infections (Week 35 of 2009 to Week 6 of 2010).

Age group	Cumulative numberof symptomatic cases/infections^a^	Number of 2009 pandemic influenza A(H1N1) cases admitted to ICUs	ICU admission rate(95% CI)(per 100,000 symptomatic cases)	ICU admission rate(95% CI)(per 100,000 infections)
0–4 years	65,544/182,067	5	7.6	2.8
			(2.5–17.8)	(0.8–6.4)
5–19 years	432,395/1,201,096	27	6.2	2.3
			(4.1–9.1)	(1.5–3.3)
20–29 years	103,280/286,890	30	29.1	10.5
			(19.6–41.5)	(7.1–14.9)
30–49 years	104,403/290,009	110	105.4	37.9
			(86.6–127.0)	(31.2–45.7)
50–64 years	53,574/148,817	74	138.1	49.7
			(108.5–173.4)	(39.1–62.4)
65+ years	11,835/32,876	41	346.4	124.7
			(248.7–469.7)	(89.5–169.1)
**Greek population**	**771,031/2,141,754**	**287**	**37.2**	**13.4**
			**(33.0–41.8)**	**(11.9–15.0)**

a Cases up to week 5/2010 – ICU admissions up to week 6/2010. Symptomatic cases were obtained based on ILI-38 definition (ILI-38: fever >38°C and cough or sore throat).

A total of 130 deaths were recorded during the same period. The estimated CFR was 6.3 deaths/100,000 infected cases and 17.5 deaths/100,000 symptomatic cases (**Table 3**). CFR estimates were markedly higher among individuals over 50 years of age (77 and 313 deaths per 100,000 symptomatic cases aged 50–64 and ≥65 years old, respectively). Individuals older than 65 years old had 185 times higher risk of death compared to individuals 5–19 years old, if infected.

**Table 3:** Overall and Age-Specific Case Fatality Rate per 100,000 symptomatic cases and per 100,000 infections (Week 35 of 2009 to Week 6 of 2010).

Age group	Cumulative numberof symptomatic cases/infections^a^	Number of 2009 pandemic influenza A(H1N1) deaths	CFR(95% CI)(per 100,000 symptomatic cases)	CFR(95% CI)(per 100,000 infections)
0–4 years	62,357/173,213	1	1.60	0.58
			(0.04–8.93)	(0.01–0.32)
5–19 years	414,362/1,151,005	7	1.69	0.61
			(0.68–3.48)	(0.25–1.25)
20–29 years	101,695/282,485	11	10.8	3.89
			(5.40–19.4)	(1.94–6.97)
30–49 years	101,772/282,700	34	33.4	12.03
			(23.1–46.7)	(8.33–16.81)
50–64 years	52,064/144,623	40	76.8	27.66
			(54.9–104.6)	(19.76–37.66)
65+ years	11,835/32,876	37	312.6	112.54
			(220.2–430.7)	(79.25–155.09)
**Greek population**	**744,085/2,066,902**	**130**	**17.5**	**6.29**
			**(14.6–20.8)**	**(5.25–7.47)**

a Cases up to week 4/2010 – deaths up to week 6/2010. Symptomatic cases were obtained based on ILI-38 definition (ILI-38: fever >38°C and cough or sore throat).

### Disease pyramid

**Figure 4:**
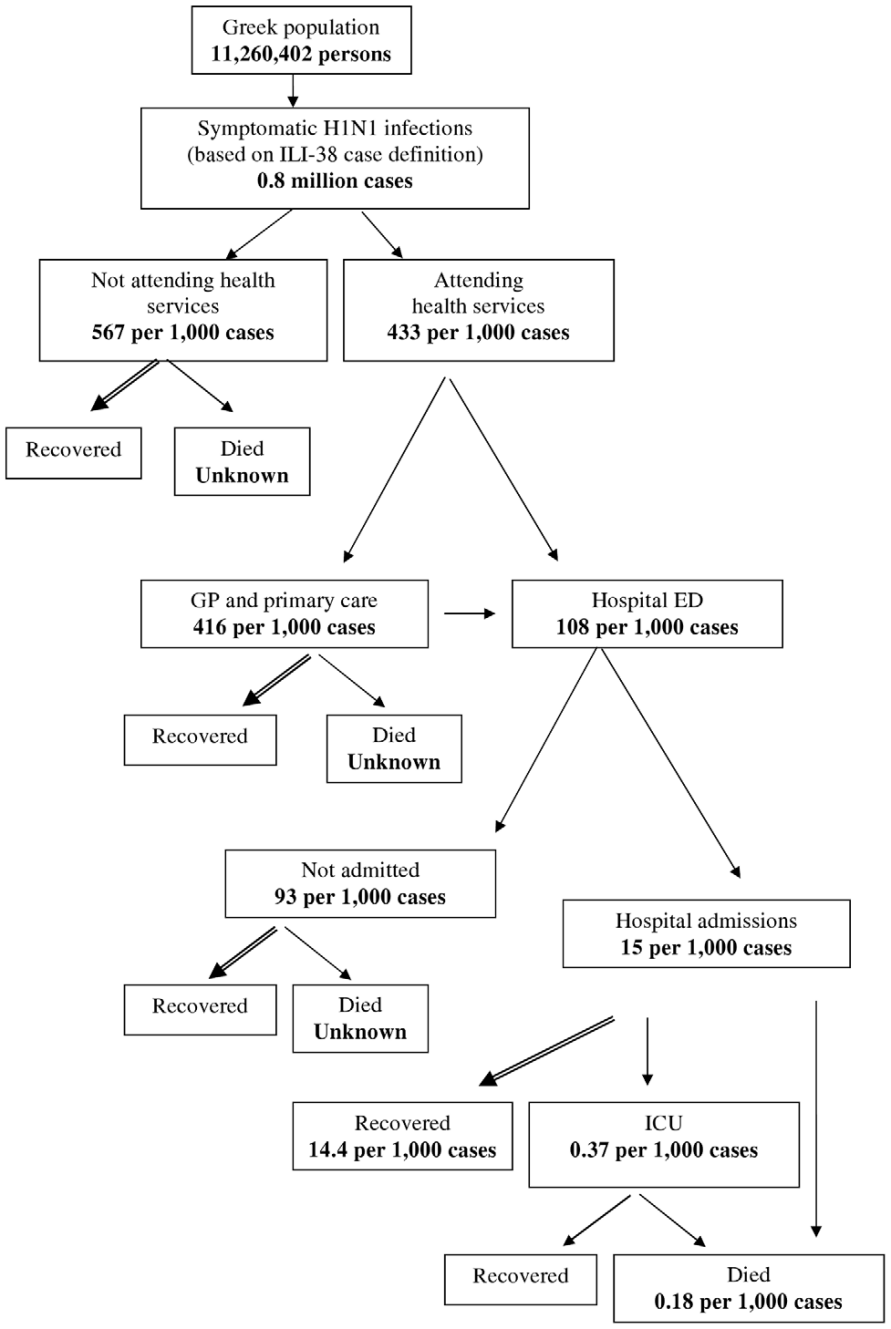
The burden of the 2009 pandemic influenza A(H1N1) pandemic in the Greek population up to week 6 of 2010. Estimates concerning the proportion of symptomatic cases seeking medical care from their GPs/hospital emergency departments and being hospitalized were based on data collected from the weekly phone surveys. The proportion of cases admitted to ICUs or dying during hospitalization was obtained from surveillance data. Symptomatic H1N1 case estimates were based on ILI-38 definition (ILI-38: fever >38°C and cough or sore throat).

A representation of the disease pyramid, i.e. of the proportion of influenza A(H1N1) infected cases seeking medical care, requiring hospitalization, ICU admission, or dying is shown in **Figure 4**. It was estimated that 416 per 1,000 symptomatic cases visited GPs/primary care physicians and 108 per 1,000 cases visited hospital emergency departments (data obtained from phone surveys; see **Supporting Information S2**). Only a small proportion were admitted to hospitals (15 per 1,000 symptomatic cases) or, subsequently, to an ICU (0.37 per 1,000 symptomatic cases). For every 100,000 symptomatic cases, 18 deaths occurred.

## Discussion

Based on the data collected from repeated cross-sectional households surveys for 25 consecutive weeks during the autumn-early winter wave of influenza A(H1N1) in Greece in 2009–2010, it was possible to reconstruct the incidence of ILI and influenza A(H1N1) cases over time in the different age population groups. It was estimated that 7%–16% of the Greek population experienced symptomatic influenza A(H1N1), depending on ILI case definition, and that approximately one fifth of the population was infected, either symptomatically or asymptomatically. The estimated CAR is in accordance with the estimates provided for New Zealand (7.5%) [13], Reunion Island (12.9%) [26] and Singapore (5.4%–11%) [27] after the major H1N1 wave was complete (**Table 4**). It compares with the estimates provided by two telephone surveys in New York City during the initial outbreak in May-June 2009 (7.8%–12.2%) [10] though it is lower than the overall clinical attack rate derived by CDC estimates for the United States by week 6 of 2010 (14% to 28%) [28]. The infection attack rate of 19.7% estimated for Greece is similar to the estimates reported for New Zealand [29] and France [30] and lower than the estimate provided by a serological study in Hong-Kong limited to individuals 5–59 years old (10.7%) [8].

**Table 4:** Comparison of estimated attack rates, ICU admission rates and CFR of 2009 pandemic influenza A(H1N1) with other studies.

Area (Reference)	ClinicalAttack Rate	InfectionAttack Rate^f^	ICU admission rate (per 100,000 symptomatic cases)	ICU admission rate (per 100,000 infections)	CFR(per 100,000 symptomatic cases)	CFR(per 100,000 infections)
Greece	7.1%–15.6%	19.7%	37.2	13.4	17.5	6.3
Hong-Kong [8] a	-	10.7%*	-	17.6	-	4.4
New Zealand [13]	7.5%	-	-	-	5	-
New Zealand [29]	-	18.3%*	-	-	8.2	4.5
Reunion Island [26]	12.9%	-	23	-	7	-
Singapore [27]	5.4%–11%	-	-	-	6.7	-
UK [9]	-	-	-	-	40	-
UK [31]	-	1.2%–70.2%^b*^	-	-	-	-
USA [28] ^c^	14%–28%		-	-	10–42	-
USA (New York) [10] ^d^	7.8%–12.2%	-	21–34	-	5–9	-
USA (Milwaukee and New York) [33] e	-	-	28–239	-	7–48	-
France [30]	-	18.1%	-	-	-	-

a Assessed in individuals 5–59 years old ^b^Depending on age and region ^c^April 2009- February 2010 ^d^May–June 2009.

e April–July 2009 ^f^Estimates denoted with *were obtained by serological surveys.

Clinical attack rates were found to vary considerably according to age, with school children/adolescents representing the most affected population group (27%–54%) and individuals older than 64 years being the least affected (0.6%–2%). Similarly, the estimated infection attack rates ranged from 1.6% in individuals older than 64 years to 76.2% in children 5–19 years old. The age-specific estimates provided for the United Kingdom and Hong-Kong from large serological studies [7], [8], [31] confirm the age-specific pattern of influenza ARs identified in our study. The UK serological study confirms our finding of high infection attack rates among school children/adolescents (IAR: 70% among children 5–14 years in UK as compared to 76.2% among children/adolescents 5–19 years in Greece) [31]. The pattern of the age-specific attack rates in this pandemic was similar to that observed in the 1957 influenza pandemic [32].

Estimates of the ICU admission rate and death rate in the Greek population have been provided by a recent study [25]. The ascertainment of the number of H1N1 cases allowed to further estimate the ICU admission rate and the CFR per symptomatic cases as well as per infected cases. Data on the ICU admission rate are limited. Our estimate was 37.2 ICU admissions per 100,000 symptomatic cases and is consistent with the findings of other studies (**Table 4**). In New York City, 21–34 ICU admissions were estimated to occur per 100,000 cases (10). An analysis of data from two US cities provided a range of 28–239 ICU admissions per 100,000 cases [33] and in Reunion Island the corresponding rate was 23 ICU admissions per 100,000 cases [26]. Our estimate of ICU admissions per 100,000 infected cases was 13.4, slightly lower compared to the estimate of 17.6 reported by a study in Hong-Kong that combined epidemiologic surveillance data with serologic data [8].

The estimated CFR in the Greek population was 17.5 deaths per 100,000 symptomatic cases. In the United Kingdom [9], the CFR was estimated to be 40 deaths per 100,000 symptomatic cases (**Table 4**). Similarly, according to the estimated number of deaths and cases provided by the CDC, the CFR is calculated at approximately 10–42 deaths per 100,000 cases for USA [28]. The rates reported for New Zealand, Reunion Island and Singapore were 5–8, 7 and 7 deaths per 100,000 cases, respectively [13], [26], [27], [29]. In Hong-Kong, the CFR among infected individuals 5–59 years old was estimated 4.4 deaths per 100,000 infections, close to the estimate of 6.3 obtained from our data [8]. Potential underestimation in our study may have arisen because deaths occurring outside the hospital setting, as shown in **Figure 4**, may not be adequately recognized and reported. Another reason would be attribution of death to other chronic diseases (e.g. cardiovascular or chronic lung disease) when in fact undiagnosed influenza was the cause of exacerbation and death. However, a common finding of all these studies is that the CFR was substantially lower than that observed in the previous pandemics of the twentieth century. This could be largely attributed to improvements in health care such as the availability of antiviral treatment, advances in intensive care medicine and treatment of bacterial super-infections. It should be noted that, despite the low CFR, the 2009 pandemic influenza A(H1N1) had a substantial health burden in terms of years of life lost as many deaths were in children, although probably not as high as estimated in one recent study [34] since that study failed to adjust for serious underlying health conditions in many of the children who died from H1N1.

In this analysis, the number of 2009 H1N1 cases was estimated as a proportion of ILI cases. Although this approach is commonly used, it presents two major challenges. The first challenge is ILI case definition. Although ILI case definitions in different countries present considerable heterogeneity [35], fever is usually included with a temperature cutoff set to 37.8°C to 38.0°C. However, in a meta-analysis of volunteer challenge studies, only 34.9% of volunteers had fever >37.8°C [22] and 66.9% experienced at least one symptom. In a recent Chinese study, 36% of RT-PCR-confirmed H1N1 cases experienced fever >38°C, and a substantial proportion (31.5%) had temperature between 37.3–38.0°C [23]. In the current study, the questionnaire allowed detailed data collection on each symptom and modified definitions of ILI were used to obtain estimates for the number of influenza-like illness and H1N1 symptomatic cases. A second challenge in estimating the number of H1N1 cases from ILI data is that it is usually done based on reported ILI surveillance. However, not all ILI cases seek consultation. In our study, ILI rates in the general population were directly estimated through weekly cross-sectional phone surveys in Greek households. Thus the presented figures are intuitively closer to the real truth and a selection bias that could relate to more severe symptomatology leading to consultation by a physician is minimized.

The rationale behind this study of phone interviews in households is similar to that of surveys of families in landmark papers [36]–[38] where valuable data were obtained on the age pattern of influenza CARs through household surveys in which family members were queried about acute respiratory illnesses. Furthermore, telephone surveys, assessing influenza-like illness in US during 2006, were found to provide results consistent with surveillance data [19]. Our study is however subject to a number of limitations. First, only households with landline phones were included in the survey. This may have resulted in an under-representation of extremely poor households where there is no landline phone. Second, to derive estimates for 2009 pandemic influenza A(H1N1) incidence, we used the proportion of samples tested positive out of the total number of samples sent to laboratories. However, people from whom samples were taken may diverge from people reporting ILI/ARI in the telephone survey, particularly at a time when mitigation strategies were recommended (in Greece, 15 July 2009 onwards). The impact of that could be a possible inflation of the estimated clinical attack rates in the population as laboratory testing is normally performed in patients with more severe symptoms, of which a greater fraction are associated with influenza. However, it should be noted that in Greece the number of samples sent for testing was very high relative to the population size (approximately 44.300 samples tested by week 6/2010) which suggests that laboratory testing was applied widely. Third, the total number of infections was obtained by dividing the number of symptomatic cases by 0.36, i.e. the estimated proportion of cases experiencing fever. The same proportion was applied to all age groups. Although Cao et al. [23] report no significant age difference among febrile and afebrile cases, it might be argued that the proportion of febrile cases may differ by age of infected individuals. In that case, the number of infections by age cannot be estimated precisely. Fourth, it is possible that some of the household representatives may have failed to correctly report the occurrence of influenza-related symptoms for each household member during the previous week (recall bias). It is not clear, though, whether this might have introduced systematic bias. The time proximity of the recall period and the survey is anticipated to have reduced this bias. Similar biases may have been present in household surveys aimed at assessing the burden of disease in previous pandemics. Finally, despite the surveillance effort to collect data on influenza A(H1N1) ICU admissions and deaths, under ascertainment and underreporting may occur. In particular, the number of deaths related to A(H1N1) might have been underestimated because there may have been deaths occurring outside the hospital setting that have not been identified.

In conclusion, our study attempted to describe the true extent of 2009 pandemic H1N1 infection in Greece and provides insights into the age-specific spread and severity of the infection during the major autumn-early winter wave in Greece. These findings may contribute to an overall assessment of the impact of 2009 pandemic influenza A(H1N1) as well as to the preparedness for future influenza pandemics or other emerging infectious diseases.

## Supporting Information

Figure S1 **Age-specific incidence of influenza-like illness (ILI) and acute respiratory illness (ARI) per 100,000 population per week in Greece (3-week weighted moving average).** (ILI-38: fever >38°C and cough or sore throat, ILI-37: fever 37.1–38°C and cough or sore throat, ARI-38: any two of fever >38°C, cough, sore throat and runny nose, ARI-37: any two of fever 37.1–38°C, cough, sore throat and runny nose).
(TIF)

Figure S2 **Incidence of influenza-like illness (ILI) and acute respiratory illness (ARI) per 100,000 population per week in Greece (3-week weighted moving average).** (ILI-38: fever >38°C and cough or sore throat, ILI-37: fever 37.1–38°C and cough or sore throat, ARI-38: any two of fever >38°C, cough, sore throat and runny nose, ARI-37: any two of fever 37.1–38°C, cough, sore throat and runny nose).
(TIF)

Supporting Information S1 **Telephone survey on influenza-like illness.**
(DOC)

Supporting Information S2 **Frequency of reported symptoms and proportion of ILI cases seeking medical advice.**
(DOC)

Supporting Information S3 **Age and gender distribution of the population and average household size in Greece (data from 2001 census, Hellenic Statistical Authority).**
(DOC)

Table S1 **2009 Pandemic Influenza A(H1N1) Clinical Attack Rates based on acute respiratory illness (ARI) case definition.**
(DOC)
