# Supplementary figures and images for: Estimating the Disease Burden of 2009 Pandemic Influenza A(H1N1) from Surveillance and Household Surveys in Greece
Source: PLoS One. 2011 Jun 9;6(6):e20593. doi: 10.1371/journal.pone.0020593 (PMC3111416; doi:10.1371/journal.pone.0020593)

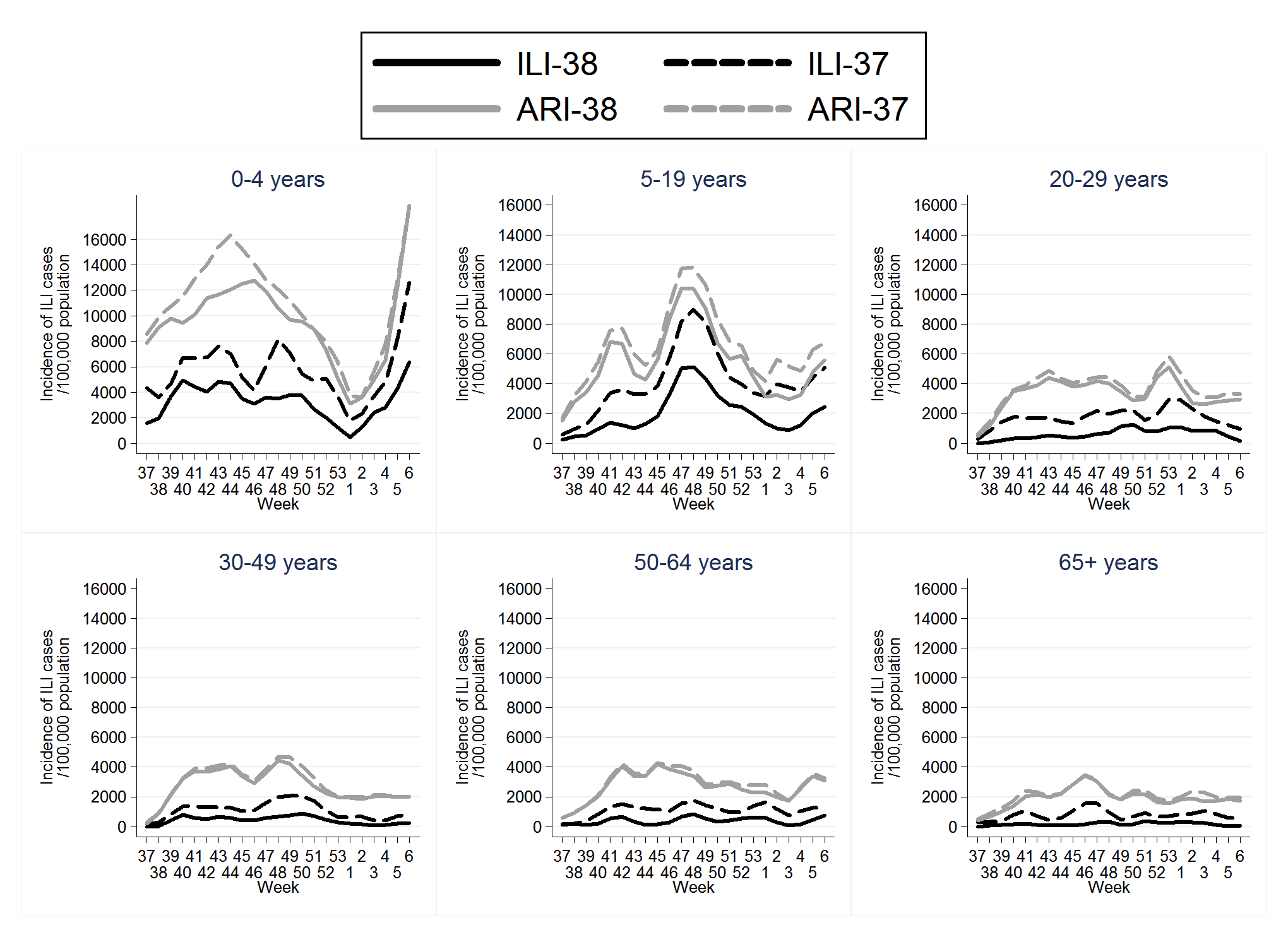

Supplement: Figure S1 — Age-specific incidence of influenza-like illness (ILI) and acute respiratory illness (ARI) per 100,000 population per week in Greece (3-week weighted moving average). (ILI-38: fever >38°C and cough or sore throat, ILI-37: fever 37.1–38°C and cough or sore throat, ARI-38: any two of fever >38°C, cough, sore throat and runny nose, ARI-37: any two of fever 37.1–38°C, cough, sore throat and runny nose). (TIF) [file pone.0020593.s001.tif]

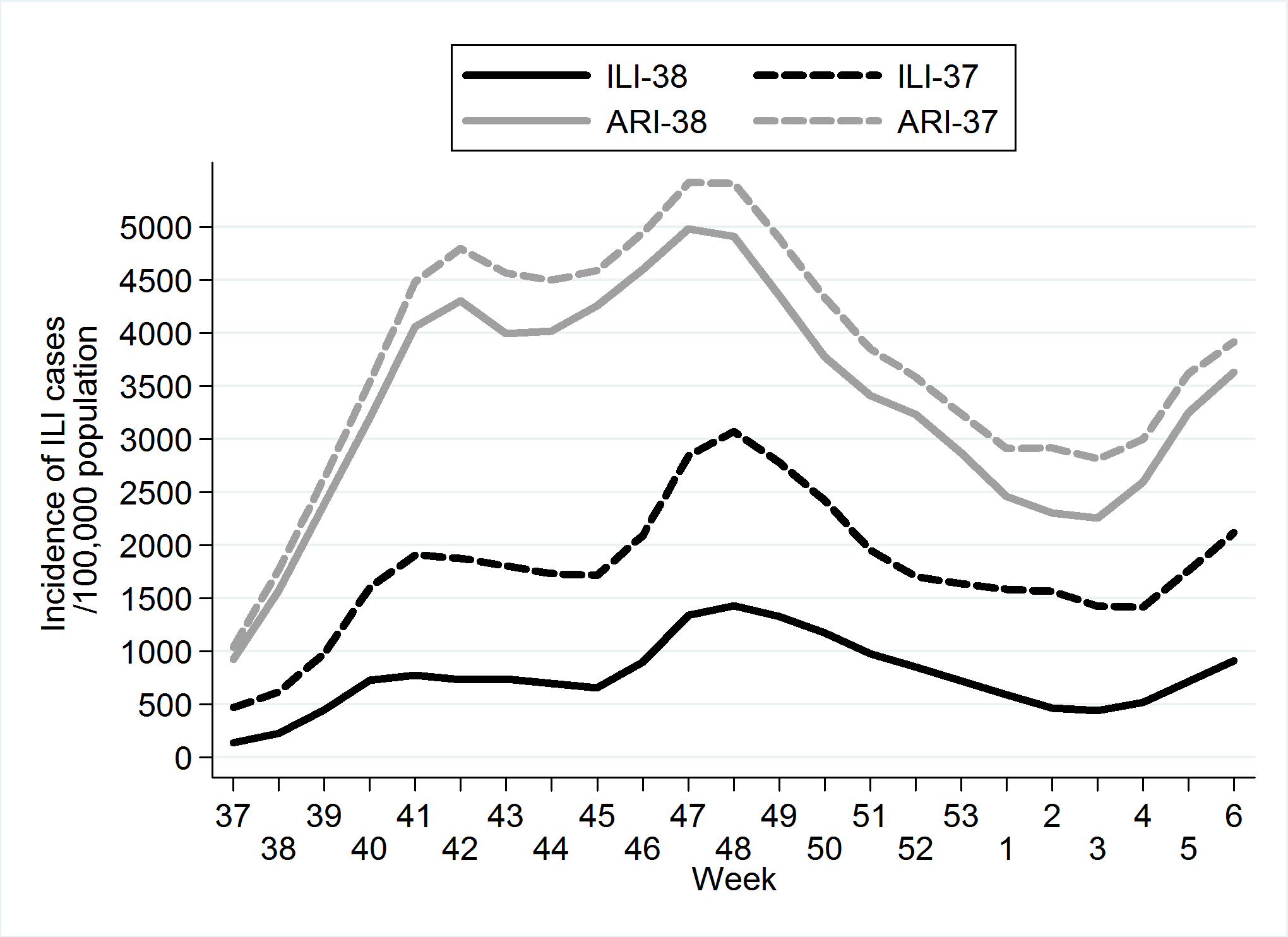

Supplement: Figure S2 — Incidence of influenza-like illness (ILI) and acute respiratory illness (ARI) per 100,000 population per week in Greece (3-week weighted moving average). (ILI-38: fever >38°C and cough or sore throat, ILI-37: fever 37.1–38°C and cough or sore throat, ARI-38: any two of fever >38°C, cough, sore throat and runny nose, ARI-37: any two of fever 37.1–38°C, cough, sore throat and runny nose). (TIF) [file pone.0020593.s002.tif]
