# Supplementary material for: Estimating the Disease Burden of 2009 Pandemic Influenza A(H1N1) from Surveillance and Household Surveys in Greece
Source: PLoS One. 2011 Jun 9;6(6):e20593. doi: 10.1371/journal.pone.0020593 (PMC3111416; doi:10.1371/journal.pone.0020593)
Supplement: Supporting Information S1 — Telephone survey on influenza-like illness. (DOC) [file pone.0020593.s003.doc]

**Supporting information S1. Telephone survey on influenza-like illness**

The telephone survey has been carried out in 1,000 households per week for 25 weeks. Proportional quota sampling was used each week to ensure that selected households were representative of Greek households in general, with quotas based on household size and urban/rural location. The weekly samples of 1,000 households included in the surveys were located in Athens (main metropolitan area of Southern Greece, 40% of households), Thessaloniki (main metropolitan area of Northern Greece, 10% of households), and other urban (25%), semi-urban (10%), and rural (15%) areas. Data collection was performed during the first 2 days of each week (Monday and Tuesday) and questions on the occurrence of symptoms referred to the preceding week (Monday through Sunday). If the reported symptoms matched the definition of an influenza-like illness or acute respiratory illness, an interview was arranged with the affected member to collect data concerning the details of the symptoms, severity of disease, whether he/she sought medical care, etc

The questionnaire comprised 3 sections; section A contains questions on the number of household members, demographic characteristics, and the occurrence of influenza-related symptoms during the previous week. Section B is addressed only to members who reported influenza-related symptoms and contains questions concerning symptoms, disease onset and relevant items. Section C was variable from week to week and included questions on vaccination, knowledge/perceptions towards influenza and school closures, and was addressed to all participating households (not shown).

**SECTION Α. ALL HOUSEHOLDS**

1. How many people are there in your household?

2. Can you tell me the age and gender of each household member? (Beginning with the oldest member. For members <1 year of age, the exact number of months should be recorded).

2a. In households with females older than 15 years, is there a woman in a state of pregnancy in the household? If yes, which member?

3. Could you tell me whether any of the household members has the following diseases? (Answers are recorded for each member – multiplicity is allowed)

- 1. Chronic cardiovascular disease (coronary heart disease, angina, myocardial infarction, congestive heart failure, congenital heart disease (excluding mild hypertension)
  2. Chronic respiratory diseases (chronic bronchitis, allergic and bronchial asthma, chronic obstructive pulmonary disease, emphysema, bronchectasis, cystic fibrosis, pneumonectomy)
  3. Chronic metabolic disorders (diabetes mellitus)
  4. Sickle-cell anemia or other hemoglobinopathies (thalassaemia, splenectomy)
  5. Chronic kidney or liver disorders (chronic renal deficiency, renal syndrome, hemodialysis, liver cirrhosis, chronic hepatitis, alcoholism)
  6. Immunodeficiency due to disease or medication: organ transplantation, lymphoma, Hodgkin’s disease, leukemia, multiple myeloma, chronic intake of corticosteroids or other immunosuppressive medication, cancer, chemotherapy, HIV infection/AIDS)
  7. Chronic neurological/neuromuscular diseases (stroke, tetraplegia, paraplegia, multiple sclerosis, cochlear implants, craniocerebral injuries)
  8. None of the above
  9. I don’t know/no answer

4. During the previous week, did any household member present any of the following symptoms: fever >37 ^o^C, cough, sore throat, runny nose?

If yes, it should be recorded which member(s) had these symptoms. Ask whether fever was above 38 ^o^C. For each of the members experiencing at least one symptom, ask the following questions:

1. Did he/she have to stay home because of these symptoms? If yes, for how many days?
2. Did he/she have to stay in bed and, if yes, for how many days?

- **For each member with fever >38 ^ο^C or at least 2 of the following symptoms: fever >37 ^o^C, cough, sore throat, and/or runny nose, arrange a personal telephone interview. For persons <15 years old, arrange an interview with the mother. This interview consists of the following questions:**

**SECTION B. ONLY HOUSEHOLD MEMBERS WITH SYMPTOMS**

Β1. Which of the following signs/symptoms did you experience? (multiple answers are possible)

| Fever | Headache |
| --- | --- |
| Cough | Vomiting |
| Dyspnea – shortness of breath | Wheezing |
| Fatigue/weakness | Diarrhea |
| Chill | None of the above |
| Myalgia | Other |
| Runny or stuffy nose | I don’t know |
| Sore throat |  |

B2. When did these symptoms first occur?

Β3. Were any other household members presenting with similar symptoms before you? If yes, how many members?

Β4. What is your weight (in kg) and height (in cm)?

Β5. Did you have to stay home because of these symptoms? If yes, for how many days?

Β6. Did you visit a doctor?

1. Yes
2. No
3. Didn’t answer

Β7. Did you visit a hospital?

1. Yes, but I was not admitted
2. Yes, and I was admitted
3. No
4. Didn’t answer

Β8. Did you receive Tamiflu?

1. Yes
2. No
3. Don’t know/didn’t answer

Β9. Did you take antibiotics?

1. Yes
2. No
3. Don’t know/Didn’t answer
