# Supplementary material for: Estimating the Disease Burden of 2009 Pandemic Influenza A(H1N1) from Surveillance and Household Surveys in Greece
Source: PLoS One. 2011 Jun 9;6(6):e20593. doi: 10.1371/journal.pone.0020593 (PMC3111416; doi:10.1371/journal.pone.0020593)
Supplement: Supporting Information S2 — Frequency of reported symptoms and proportion of ILI cases seeking medical advice. (DOC) [file pone.0020593.s004.doc]

**Supporting information S2. Frequency of reported symptoms and proportion of ILI cases seeking medical advice**

One participant per household was asked to report on the occurrence of influenza-related symptoms for each household member. A personal phone interview was arranged with household members experiencing either fever >38 ^o^C or any two of the following: fever 37.1-38 ^o^C, cough, sore throat, runny nose. The aim of this interview was to collect data concerning details of the symptoms, severity of disease, whether the affected member sought medical care and other relevant items..

During week 35 of 2009 and week 6 of 2010, there were 1,571 individuals in the contacted households who had experienced ILI symptoms and agreed to provide additional information in a personal phone interview. Their responses concerning detailed symptoms and visits to GPs/hospitals are shown below in Tables A and B, respectively.

1. **Frequency of reported symptoms**

| **Symptom** | **n (%)** |
| --- | --- |
| Fever | 814 (51.8) |
| Cough | 1219 (77.6) |
| Dyspnea – shortness of breath | 229 (14.6) |
| Fatigue/weakness | 560 (35.7) |
| Chill | 309 (19.7) |
| Myalgia | 386 (24.6) |
| Runny or stuffy nose | 1077 (68.6) |
| Sore throat | 964 (61.4) |
| Headache | 544 (34.6) |
| Vomiting | 99 (6.3) |
| Wheezing | 182 (11.6) |
| Diarrhea | 110 (7.0) |
| Other | 17 (1.1) |

1. **Proportion of ILI cases seeking medical advice**

|  | **% (95% CI)** |
| --- | --- |
| Visit to GPs/primary care | 41.6 (38.6, 44.7) |
| Visit to hospital | 10.8 (8.7, 12.9) |
| - Not admitted | 9.3 (7.4, 11.2) |
| - Admitted | 1.5 (0.6, 2.4) |
| Visit to GP or primary care or hospital | 43.3 (40.3, 46.4) |
