# Supplementary material for: Estimating the Disease Burden of 2009 Pandemic Influenza A(H1N1) from Surveillance and Household Surveys in Greece
Source: PLoS One. 2011 Jun 9;6(6):e20593. doi: 10.1371/journal.pone.0020593 (PMC3111416; doi:10.1371/journal.pone.0020593)
Supplement: Supporting Information S3 — Age and gender distribution of the population and average household size in Greece (data from 2001 census, Hellenic Statistical Authority). (DOC) [file pone.0020593.s005.doc]

**Supporting information S3. Age and gender distribution of the population and average household size in Greece (data from 2001 census, Hellenic Statistical Authority)**

Age and gender distribution of the population and average household size in Greece (data from 2001 census, Hellenic Statistical Authority)

A. Age distribution of the Greek population [1]

| **Age group** | **N** | **%** |
| --- | --- | --- |
| 0-14 | 1,664,085 | 15.2 |
| 15-24 | 1,565,320 | 14.3 |
| 25-64 | 5,903,075 | 53.8 |
| 65+ | 1,831,540 | 16.7 |

B. Gender distribution of the Greek population [1]

| **Gender** | **N** | **%** |
| --- | --- | --- |
| Males | 5,427,682 | 49.5 |
| Females | 5,536,338 | 50.5 |

C. Average household size (from the 2001 census) [2]

| **Number of households** | **Members** | **Average**  **household size** |
| --- | --- | --- |
| 3,674,381 | 10,291,186 | 2.8 |

**References:**

1.http://www.statistics.gr/portal/page/portal/ESYE/BUCKET/A1604/Other/A1604_SAP01_TB_DC_00_2001_02_F_EN.xls

2.http://www.statistics.gr/portal/page/portal/ESYE/BUCKET/A1604/Other/A1604_SAP05_TB_DC_00_2001_10_F_EN.xls)
