# Supplementary material for: Estimating the Disease Burden of 2009 Pandemic Influenza A(H1N1) from Surveillance and Household Surveys in Greece
Source: PLoS One. 2011 Jun 9;6(6):e20593. doi: 10.1371/journal.pone.0020593 (PMC3111416; doi:10.1371/journal.pone.0020593)
Supplement: Table S1 — 2009 Pandemic Influenza A(H1N1) Clinical Attack Rates based on acute respiratory illness (ARI) case definition. (DOC) [file pone.0020593.s006.doc]

**Table S1.** 2009 Pandemic Influenza A(H1N1) Clinical Attack Rates based on acute respiratory illness (ARI) case definition

| **Age group** | **Clinical Attack Rate of Influenza A(H1N1)** | |
| --- | --- | --- |
|  | **% (95% CI)** | |
|  | **Based on the number of :** | |
|  | **ARI-38^a^ cases** | **ARI-37^b^ cases** |
| 0–4 years | 41.2 | 45.4 |
|  | (25.5-56.9) | (28.6-62.2) |
| 5–19 years | 67.1 | 80.2 |
|  | (43.3-91.0) | (53.6-100.0) |
| 20–29 years | 33.5 | 37.3 |
|  | (23.0-44.1) | (25.3-49.3) |
| 30–49 years | 19.5 | 21.0 |
|  | (13.0-26.0) | (13.9-28.1) |
| 50–64 years | 16.4 | 17.9 |
|  | (11.3-21.5) | (12.2-23.6) |
| 65+ years | 5.5 | 6.0 |
|  | (3.2-7.8) | (3.5-8.4) |
| **Greek population** | **26.1** | **29.5** |
|  | **(20.8-31.4)** | **(23.4-35.6)** |

Overall and Age-Specific 2009 Pandemic Influenza A(H1N1) Clinical Attack Rates (depending on the definition of acute respiratory illness (ARI) ((Week 35 of 2009 to Week 6 of 2010)

^a^ARI-38: any two of fever >38^o^C, cough, sore throat and runny nose, ^b^ARI-37: any two of fever 37.1-38^o^C, cough, sore throat and runny nose.
